# Numerical simulations on compression behaviors of the laminated shale based on the digital image technology and the discrete element method

**DOI:** 10.1038/s41598-024-66333-1

**Published:** 2024-07-10

**Authors:** Zidong Wang, Xiaoxuan Ding, Jianlin Liu, Li-Yun Fu

**Affiliations:** 1 College of Pipeline and Civil Engineering, China University of Petroleum (East China), Qingdao, 266580 China; 2 College of Geosciences, China University of Petroleum (East China), Qingdao, 266580 China

**Keywords:** Laminated shale, Microscopic layered structures, Digital core, Discrete element method, Parallel bonding model, Geophysics, Computational science

## Abstract

As an unconventional reservoir sedimentary rock, shale contains a series of layers and various microstructures that lead to complex mechanical properties, such as the anisotropy of stiffness and strength. This study is directed towards the anisotropy caused by the microstructures of the shale, employing the 2D particle flow code (PFC^2D^) to explore stiffness, strength, failure mode, and micro-crack evolution. More realistic microstructures and the calibration of microscopic parameters of the shale are reasonably considered through the computed tomography (CT) images and mineral analysis. The corresponding numerical simulation results are fully compared with the experimental results. In what follows, the sensitivity analysis is conducted on the key microscopic parameters and microstructure characteristics in numerical samples with laminated characteristics. The results show that the influence of microscopic parameters of the parallel bonding model on macroscopic parameters is related to the layering angle and the face type, and the microstructures and initial cracks of numerical samples can considerably affect the macroscopic mechanical behaviors of the laminated samples. Next, the effect of confining pressure on the mechanical properties of layered shale is also discussed based on the numerical results. These findings highlight the potential of this approach for applications in micro-scaled models and calibration of microscopic parameters to probe mechanical behaviors of the laminated rock.

## Introduction

In recent years, with the increasing demand of fossil energy in the world, horizontal drilling and hydraulic fracturing technologies which are typical cases of engineering practice surpassing theoretical research have been widely applied to the extraction of unconventional reservoirs, in particular oil and gas shales^1^. Theoretical studies on the mechanical properties of shales can provide theoretical guidance of drilling and hydraulic fracturing, thereby improving the efficiency of shale gas extraction^2^. Compared to traditional reservoir rocks such as the sandstone and the carbonate, the shale is composed of multiple mineral components and it has more complex microscopic structural characteristics, which result in strong heterogeneity, increasing the difficulty of predicting the overall mechanical properties of the reservoir shale^3,4^. Experiments show that the proportion of minerals in shales can significantly affect the macroscopic mechanical parameters, such as the stiffness and strength of shale^5^. It is judged that, the layered structure, interlayer weak planes, and joint fractures formed by gravity during shale diagenesis can lead to the anisotropy of shales.

Recently, many experimental studies on anisotropic rocks such as the shale have been extensively conducted, which can be mainly divided into two aspects, including the stiffness and the strength. In terms of the stiffness of shale, different types of shales^6–8^ with different bedding angles were tested by the triaxial compression experiment and the ultrasonic measurements, in order to obtain the stiffness of samples with different layered angles under different confining pressures. The results indicate that the stiffness of shales with different mineral compositions are significantly different, and the anisotropy of stiffness is also affected by the distribution of layered structures. For the strength of shale, there have been numerous studies on triaxial compression experiments^9–12^, Brazilian splitting tests^8,13,14^ and direct shear tests^15–17^ on shales. From these experimental results, it can be summarized that the strength of the sample is significantly influenced by the layered angle. In the conventional triaxial compression test, the compressive strength of the sample shows a U-shaped distribution as the layered angle increases, i.e., the compressive strength of the sample is relatively small when the layered angle is between 30° and 60°^18^. However, due to the strong heterogeneity and complex microstructures of shales, it is difficult to quantitatively analyze the impact of these microscopic structures on the mechanical properties of shale, directly through the experiment. In addition, compared with homogeneous rocks, experimental results of laminated shales with differentiation of mineral compositions and microstructures are much more dispersed which means higher costs for more experiments.

Therefore, numerical sample of rock based on the discrete element method (DEM), with controllable mineral compositions and microstructures, is normally used to analyze various properties of rocks^19^. By using this strategy, one can conduct multiple numerical tests with different external responses on the same microscopic structural sample. Among others, the particle -flow code (PFC), as a common program of DEM, has been widely used to study the mechanical properties of anisotropic or heterogeneous rock materials^20^.

In the DEM study of anisotropy and heterogeneity in laminated rocks, Park and Min^21^ embedded the smooth joint (SJ) model into the DEM rock matrix composed of the parallel bond (PB) model. The so-call Synthetic Rock Mass (SRM)^22^ was proposed to simulate shale samples with weak bedding planes, and it was pointed out that the SJ model can be used to simulate the anisotropic behavior of rocks caused by weak cohesion planes between bedding planes^23^. Yang et al.^14,24^ further introduced the Gaussian distribution into the SRM to create joint structures with unequal distances. However, Ramos et al.^6,7^ observed that the directionally distributed heterogeneous components also lead to strong anisotropy of stiffness and strength for the shale sample, where the CT images of the samples before and after the triaxial experiment were given. Some researchers^25,26^ analyzed the anisotropy of heterogeneous laminated rocks by setting different microscopic mechanical parameters of PB model in different components. In addition, several DEM studies^27–29^ considering the heterogeneity of granite have been well developed, and a typical example is one mature method called the Grain Based Model (GBM). In order to accurately describe the heterogeneity of granite, Shi et al.^30^ applied CT images of granite to the DEM and analyzed the impact of different mineral composition ratios on the sample’s strength. In fact, the method combining digital images with DEM has been widely reported in studies of heterogeneous materials such as concrete^31^ and composite materials^32^, but there is still a lack of report on the properties of shales. It can be concluded that, most existing works on DEM of shales have ignored the heterogeneity and microscopic structures of the material. The micro-structural difference between the ideal geometric configuration and the CT image may significantly affect the selection of microscopic parameters in the DEM simulation. As a consequence, in the present study, a series of CT images that can reflect the microscopic characteristics have been applied to the construction of DEM model on the laminated shale.

The remainder of this article is organized as follows. In “Mineral identification and the construction of numerical model” section, a set of CT images of shale samples extracted from the 3D digital core are well described and processed into binary images for the establishment of the DEM numerical samples. In “Calibration of microscopic mechanical parameters of the laminated shale” section, the main microscopic mechanical parameters applied in the numerical model of laminated shale are introduced, calibrated, and discussed with the experimental results. In “Numerical simulation on mechanical behaviors of the laminated shale” section, a series of numerical results containing different micro-structures and microscopic parameters are used to discuss the effects of component distribution, microscopic parameters, and confining pressure on the mechanical properties of laminated shale.

## Mineral identification and the construction of numerical model

### Slice images of the microstructures of shale samples

The CT data information of Mancos shale published by Ramos et al.^6,7^ in the Digital Rock Portal^33^ is used to extract the geometric properties of laminated shale throughout this work. This set of data includes a total of 9 samples with the layering angle between the drilling direction and the bedding direction being 0° (horizontal), 45°, and 90°. All of the samples are cylindrical specimens with the diameter being *d* = 25 mm and the height being *h* = 50 mm approximately. The samples are underwent CT scans before and after the triaxial test, thus a total of 18 sets of digital core data can be obtained.

**Figure 1:**
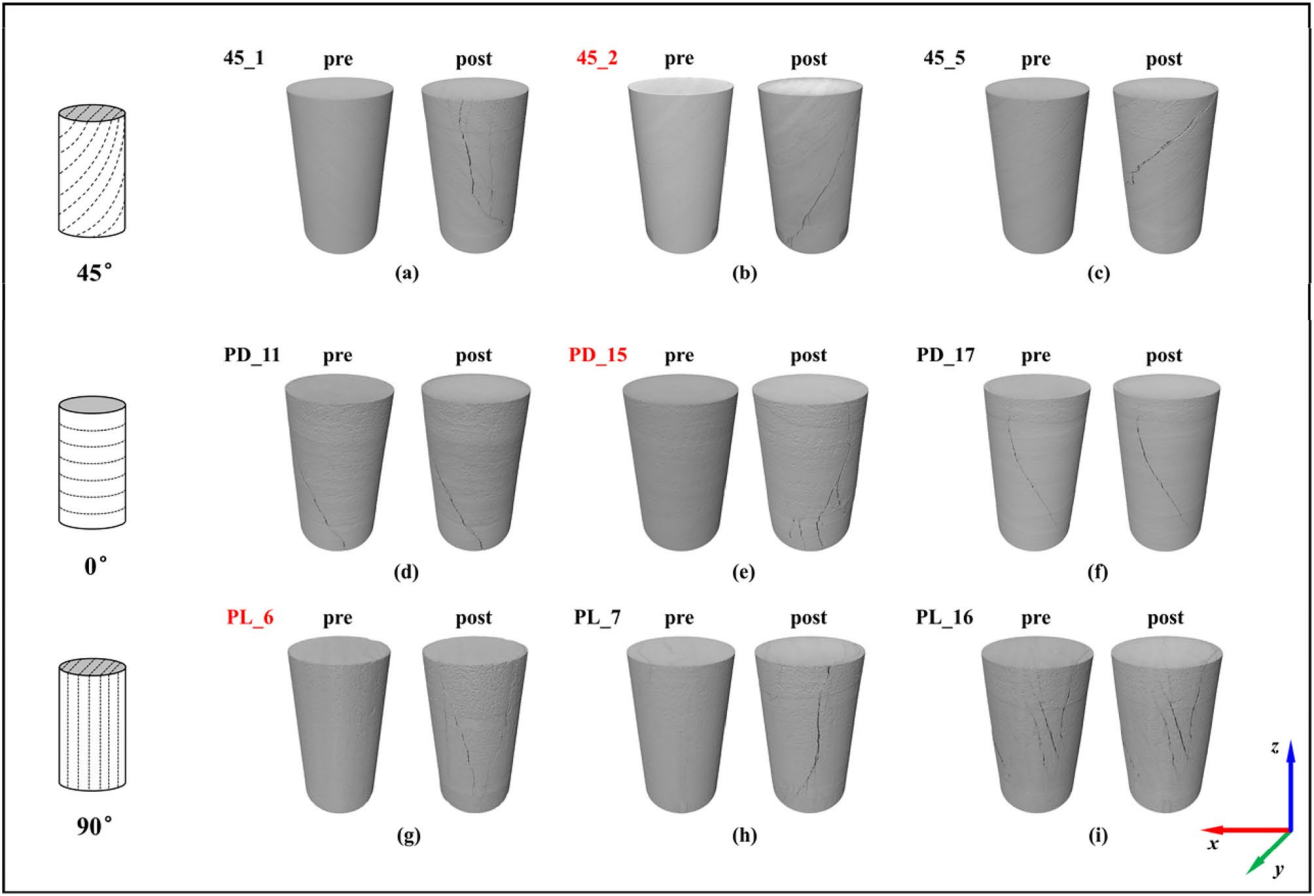
3D reconstruction of Mancos shale’s digital core, where the label of sample follows the original data in Digital Rock Portal, i.e. PL and PD denote the parallel bedding and the perpendicular bedding, respectively, and pre and post denote before and after the triaxial test, respectively.

Figure 1 shows the results of 3D reconstruction of the state of each sample before and after the triaxial test. The directions of all sample data were adjusted to make their bedding directions parallel to the *y*-axis to ensure accurate comparison of pre and post samples. Although this set of data includes 9 samples, most of the sample exist initial defects, which can be observed through the pre sample digital core. These initial defects, which are inevitable due to factors such as rock block sampling or sample cutting, can significantly reduce the stiffness and strength of the sample, and it should be considered when we investigate the impact of sample layering on rock properties. Therefore, three samples different layering angles (red labels in Fig. 1) which are equipped with the least initial defects are selected as the analysis objects.

**Figure 2:**
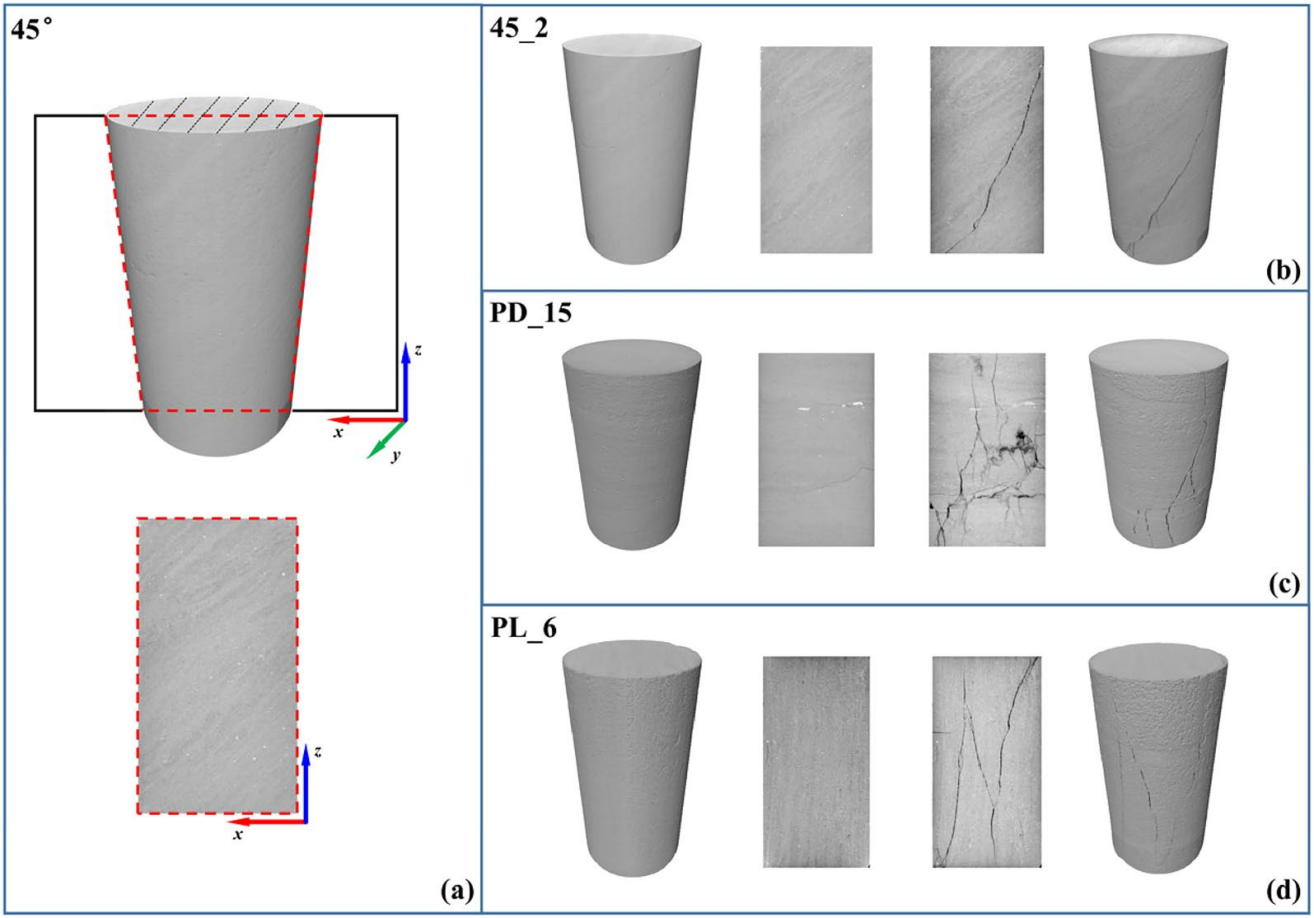
Sliced images of shale sample in the *x*–*z* plane, where (**a**) shows the process of slice, and (**b–d)** are the 3D digital cores and 2D slices before and after the triaxial compression test with the bedding angles being 0°, 45° and 90° samples.

The slicing planes for the three specimens are along the symmetric axis of the sample and parallel to the *x*–*z* plane (as shown in Fig. 2a), to fully reflect the layering characteristic of the sample. By comparing the details of shale minerals in the slice images of pre- and post-test in Fig. 2b–d, it is clearly seen that the angle adjustment of digital cores is accurate and the pre and post sliced images are at the same location. Mancos samples exhibit two major facies in their bedding (Fig. 2a), and both facies contain the same major mineral constituents, but they are in different quantities^7^. The light facies (LF) consists of approximately 52% quartz, 13% clays, 16% calcite, and 11.4% dolomite, whereas the dark facies is comprised of approximately 15% quartz, 46% clays, 5% calcite, and 19% dolomite, where the specific composition of two facies is shown in Table 2 of “Discussion on the macroscopic parameters of each single phase” section.

Compared to direct observation of the sample in previous studies^24,34^, post-test sliced images can more visually display the cracking form of the sample. According to the distribution of cracks, there are obvious oblique main cracks, with fewer secondary cracks in the 45° and 90° samples. The direction of the main crack is similar to that of the layer, but the main crack can still cross the different inner layers. However, there are many secondary micro-cracks in the 0° sample, and there are main cracks both perpendicular and parallel to the bedding plane. It is worth noting that an obvious initial crack exists in the pre-test slice image of the 0° sample, while there are no significant initial cracks in the pre-test slice images of the 0° and 90° samples.

### Image processing of the shale sample slices

The initial sliced image is converted into a grayscale image, which is actually composed of a numerical matrix ***F***_*M*×*N*_ and can be expressed as

1 $$ \left[ {F_{M \times N} } \right] = \left[ {\begin{array}{*{20}c} {f(1, \, 1)} & {f(1, \, 2)} & \cdots & {f(1, \, N)} \\ {f(2, \, 1)} & {f(2, \, 2)} & \cdots & {f(2, \, N)} \\ {} & \cdots & {f(x, \, y)} & {} \\ {f(M, \, 1)} & {f(M, \, 2)} & \cdots & {f(M, \, N)} \\ \end{array} } \right], $$

where *M* and *N* denote the total number of rows and columns of pixels in the image, respectively, and *f*(*x, y*) represents the grayscale value at the pixel point (*x, y*) in the range [0, 255].

**Figure 3:**
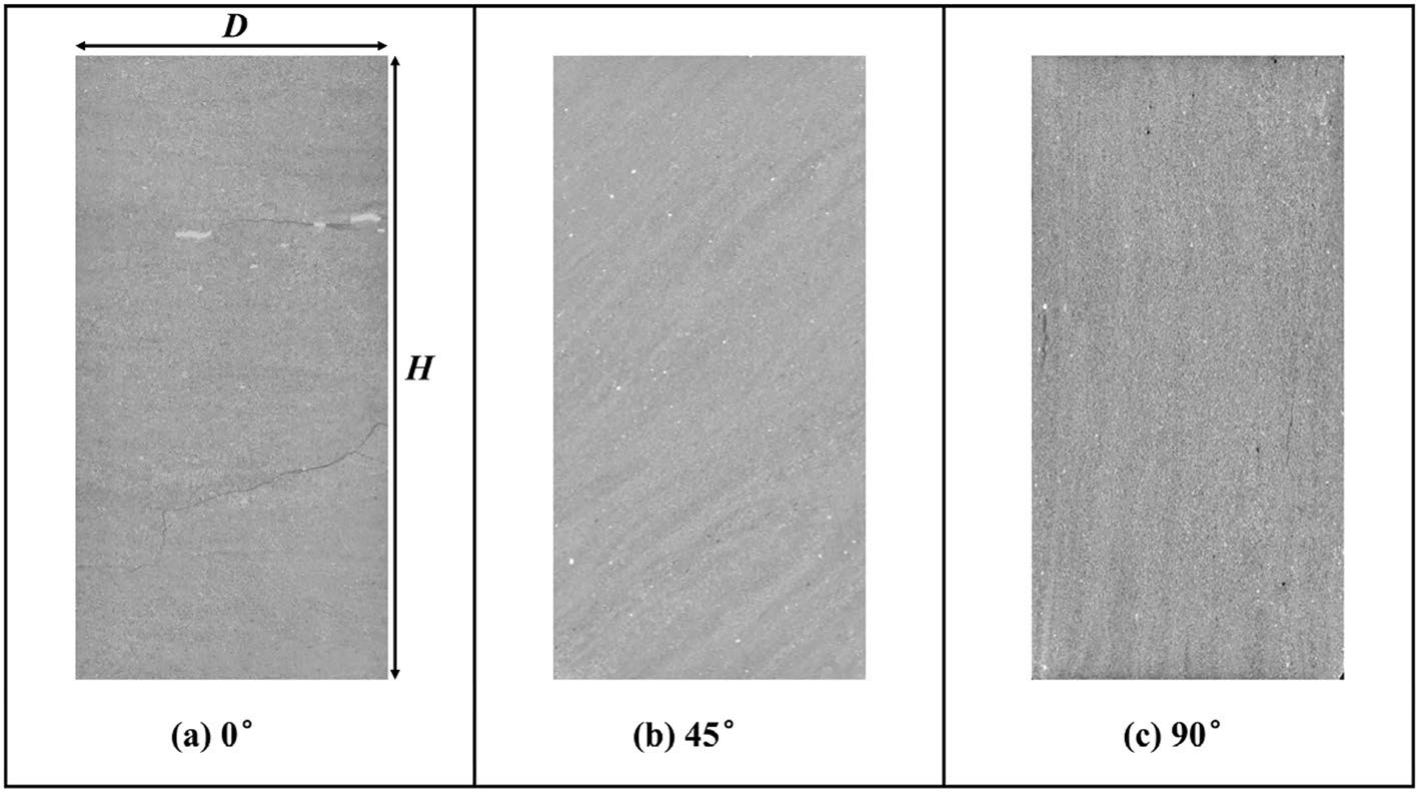
Grayscale sliced images in the *x*–*z* plane, where (**a–c**) are the slices of 0°, 45° and 90° samples, respectively.

Due to the errors in sample size and the scanning segment length, the size ratio of the initial sliced images of the different samples are inconsistent in Fig. 2b–d. In addition, lower grayscale owing to uneven lighting at the edges of sliced image also lead to errors in mineral recognition based on grayscale. Therefore, initial sliced images should be cut with their center pixel as the center to obtain the analyzed sliced images with the diameter to height ratio being 0.5. Then, moderate gradual adjustment^35^ of grayscale at the corresponding edges of each image was applied to eliminate the grayscale errors, and the resulting slice images are shown in Fig. 3. The main features of the images in Fig. 3 are the layering structure between LF and DF. Although there are still some pyrite or feldspar minerals with high grayscale values, as well as micro-cracks or pores with low grayscale values in the image, those components were correspondingly divided into LF or DF due to the low fraction.

**Figure 4:**
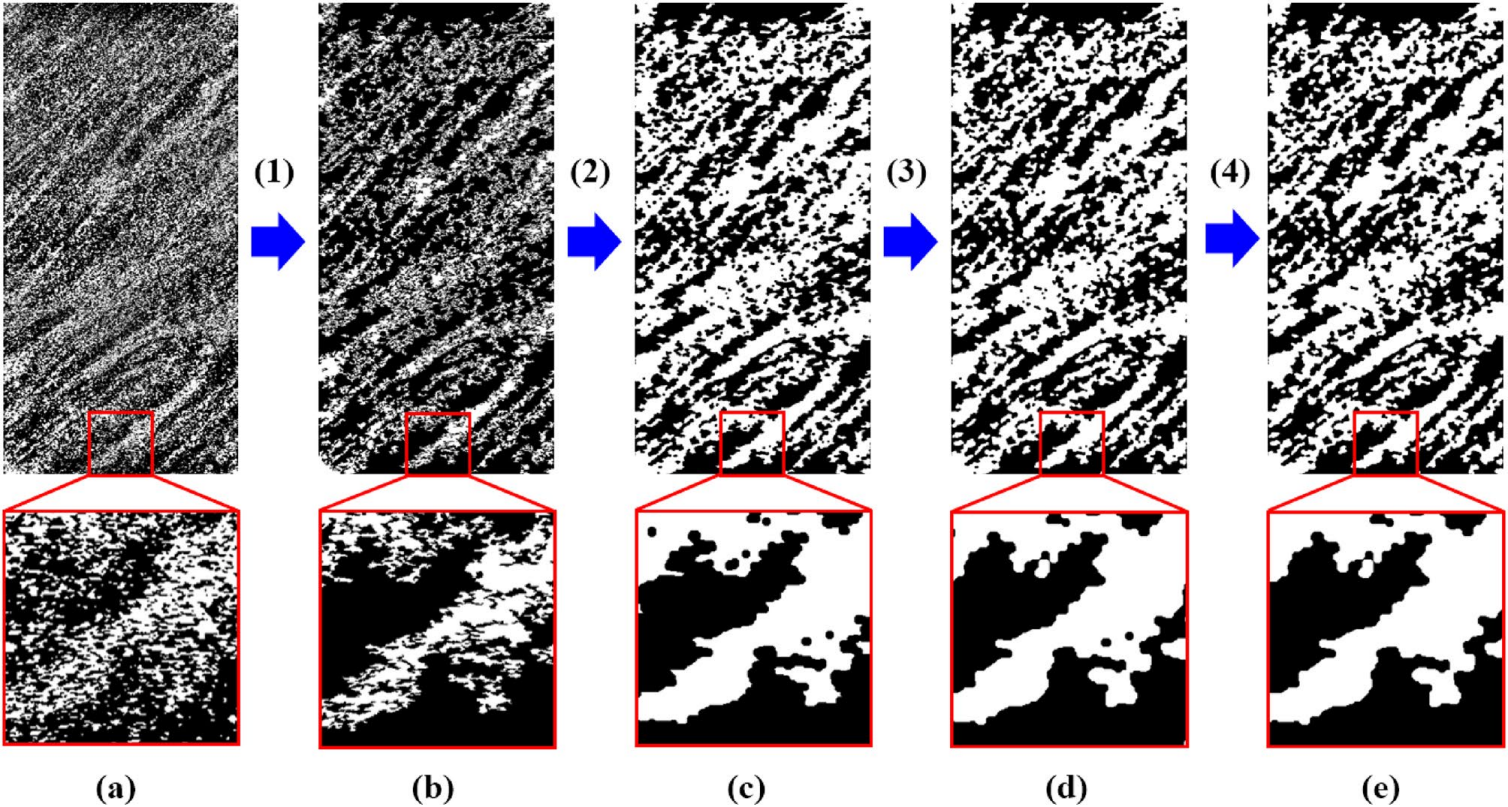
The morphological processes of sliced image of 45° sample.

Then, the threshold segmentation was applied to divide the regions of LF and DF which can convert the grayscale image into binary images. The Otsu method^36^ is the most common and reasonable way to obtain the grayscale value of threshold and as an example, the results of threshold segmentation in the 45° sample slice is shown in Fig. 4a. However, this image still needs further morphological operation^37^ in order to be applied to subsequent modeling, and the processes shown in Fig. 4 includes: (1) Remove small spots of LF and DF (by invert the image) to filter out small particles. (2) Closing operation to fill the gaps or defects in the edge of LF or DF whose structures are much smaller than those in the region of layering facies. (3) Opening operation to remove the bulges in the edge of LF or DF. (4) Remove small spots of LF and DF once more. In brief, these operations reduce the complexity of geometry on the premise of maintaining the overall layering properties which can be seen in Fig. 4e. By adjusting the corresponding parameters in morphological operation, the area ratio of LF and DF can be controlled to the value of 1:1 approximately.

### Establishment of the numerical sample

A 2D discrete element sample with equal proportions of LF and DF was established by using cellular automata simulation^38^. Well-contact disc particles evolved automatically and generated mineral microstructures to simulate the characteristics of rocks based on PFC^2D^. For the shale sample in this study, binary sliced images are used to group the generated particles for the subsequent mechanics definition. The numerical sample with the diameter *D* = 25 mm and the height *H* = 50 mm is established. It is generally believed that when the particle dimension parameter RES > 10, the influence of particle size on DEM simulation results can be ignored^39^. Based on previous simulation studies on DEM^26,30^, a total of 27,222 discs with particle radius ranging from 0.015 to 0.030 mm were generated which RES >  > 500. These parameters were chosen to ensure that the numerical model faithfully represents the heterogeneity and anisotropic properties of the shale samples.

**Figure 5:**
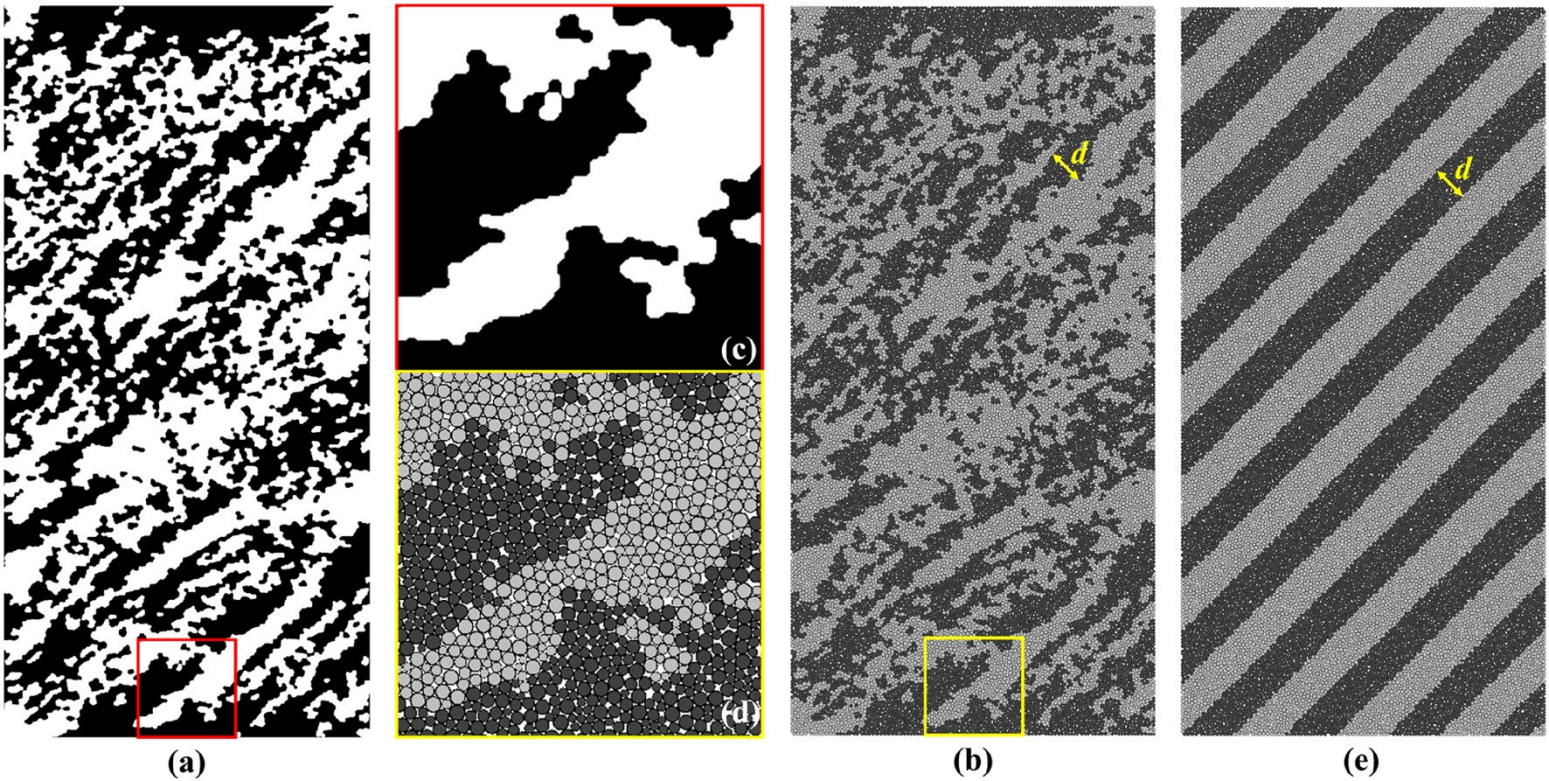
Numerical model of 45° sample, where (**a**) is the binary CT image of the 45° sample, (**b**) is the numerical discrete element CT model, (**c,d**) are the local details of binary image and numerical model, respectively, and (**e**) is the ideal numerical model with similar layered thickness.

Figure 5 shows the generation results of the 45° numerical sample. The overall distribution of mineral facies and the micro-structures in layers are well characterized using the discrete numerical model by comparing Fig. 5a–d, respectively. For convenience of subsequent comparison and discussion, the classical and ideal interbedded model^26^ should also be built with the interlayer thickness *d* = 2.5 mm, which is shown in Fig. 5e.

## Calibration of microscopic mechanical parameters of the laminated shale

### Linear parallel bond model

**Figure 6:**
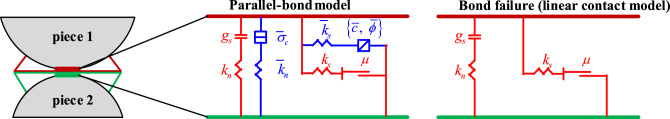
Schematic diagram of the linear parallel bond model.

The particle DEM could be used to simulate the mineral particles of rock as discs in 2D that are in contact with each other, and it can categorize the properties of discs and contacts to simulate microscopic rock minerals^21^. The linear parallel-bond model (Fig. 6) proposed by Potyondy^19,40^ is appropriate for simulating the micromechanical properties of cemented materials, such as rocks. The parallel-bond (PB) model consists of the linear contact element and the parallel-bond element as shown in Fig. 6. The linear contact element (red line in Fig. 6) is equivalent to the linear contact model which can only transmit pressure and friction-sliding and cannot resist tension and rotation. The parallel-bond element (blue line in Fig. 6) is called a parallel bond which acts in parallel with the linear contact element and can resist tension and rotation. When the parallel bond does not work, the linear parallel-bond model will degenerate into the linear contact model.

There are several main micro-mechanical parameters in the PB model, which are schematized in Fig. 6. The normal and shear stiffness of linear contact element can be respectively expressed as

2 $$ k_{{\text{n}}} { = }\frac{{AE^{*} }}{L}, $$

3 $$ k_{{\text{s}}} { = }\frac{{k_{{\text{n}}} }}{{\kappa^{*} }}, $$

where *A* is cross-sectional area between particles, i.e. *A* = 2*rt*. The other parameters are: *t* = 1 (PFC^2D^), *r* represents the contact radius of particle–particle or particle–wall where *r* = min(*R*^(1)^, *R*^(2)^) in particle–particle or *r* = *R*^(1)^ in particle–wall, *L* represents the contact distance where *L* = *R*^(1)^ + *R*^(2)^ in particle–particle or *L* = *R*^(1)^ in particle–wall. The quantity E^*^ represents the effective modulus of the linear contact element, and $\kappa^{*}$ represents the normal-shear stiffness ratio of the linear contact element. In addition, the linear contact element also includes the surface gap of contact *g*_s_ and the frictional coefficient *μ*.

Similarly, the normal and shear stiffness of the parallel-bond element can be respectively written as follows

4 $$ \overline{k}_{{\text{n}}} { = }\frac{{A\overline{E}^{*} }}{L}, $$

5 $$ \overline{k}_{{\text{s}}} { = }\frac{{\overline{k}_{{\text{n}}} }}{{\overline{\kappa }^{*} }}, $$

where $\overline{E}^{*}$ is the effective modulus of the parallel-bond element, and $\overline{\kappa }^{*}$ is the normal-shear stiffness ratio of the parallel-bond element. In addition, $\overline{\sigma }_{{\text{c}}}$ represents the tensile strength of the parallel-bond element, and $\overline{c}$ is the cohesion. The quantity $\overline{\phi }$ is the internal friction angle, and the shear strength of the parallel-bond element reads $\overline{\tau }_{{\text{c}}} = \overline{c} + \overline{\sigma }\tan \overline{\phi }$. When the value of the stress exceeds $\overline{\sigma }_{{\text{c}}}$ or $\overline{\tau }_{{\text{c}}}$, the parallel-bond element will break and it will degenerate into the linear contact model.

### Discussion on the macroscopic parameters of each single phase

Due to the lack of experimental results on each single phase, the stiffness and strength of LF and DF should be discussed firstly. Detailed mineral composition and mineral stiffness properties in these two facies were strictly confirmed by Ramos et al.^7^ using SEM and CT, which are shown in Table 1. The minerals in Table 1 are quartz, calcite, dolomite, kaolinite, illite, montmorillonite, pyrite, orthoclase, and albite. The material of clay is actually the sum of kaolinite, illite, and montmorillonite.

**Table 1:** Mineral composition and stiffness parameters of LF and DF.

Mineral	Qtz	Cal	Dol	Kaol	Ill	Smec	Pyr	Orth	Alb	Clay				
*K* (GPa)	37	70	80	46	45	9.5	140	65	55	35.5				
*G* (GPa)	41	28	45	22	20	6.9	125	30	30	17.5				

Facies	Volume fraction of each mineral (%)										Eff *K* (GPa)	Eff *G* (GPa)	Eff *E* (GPa)	Eff *μ*
LF	52.5	16.1	11.4	5.3	7.6	0.1	1.6	1.8	1.1	13	47.9	35.9	86.2	0.20
DF	14.9	4.9	19.3	10.2	30	6	4.7	1.6	3.2	46.2	46.4	27.2	68.3	0.25

The homogenization method based on the inclusion theory has been widely applied to predict the stiffness of rocks containing multiple mineral inclusions^41^. One useful method is the Mori–Tanaka (MT) method, which is efficiently used in the stiffness prediction of rocks with several kinds of mineral inclusions, i.e.

6 $$ {\overline{\mathbf{C}}} = {\mathbf{C}}_{0} + \sum\limits_{r = 1}^{N - 1} {f_{r} \left( {{\mathbf{C}}_{r} - {\mathbf{C}}_{0} } \right):{\mathbf{T}}_{r} :\left( {f_{0} {\mathbf{I}} + \sum\limits_{r = 1}^{N - 1} {f_{r} } {\mathbf{T}}_{r} } \right)^{ - 1} } , $$

where ${\overline{\mathbf{C}}}$ is the effective elastic stiffness tensor, ***C***_0_ represents the elastic stiffness tensor of the matrix, and *N* is the number of components in the shale including the matrix and (*N*–1) kinds of inclusions. The subscript *r* is the component number, where subscript 0 corresponds to the matrix. The parameter *f*_*r*_ is the volume fraction of each component, satisfying $\sum\nolimits_{r = 0}^{N - 1} {f_{r} = 1}$. The quantity ***C***_*r*_ is the elastic stiffness tensor of the *r*-th component, and ***T***_*r*_ is the tensor for orientation averaging of the *r*-th component, i.e.

7 $$ {\mathbf{T}}_{r} = \left[ {{\mathbf{I}} + {\mathbf{S}}_{r} :{\mathbf{C}}_{0}^{ - 1} :\left( {{\mathbf{C}}_{r} - {\mathbf{C}}_{0} } \right)} \right]^{ - 1} , $$

where ***S***_*r*_ is the Eshelby tensor of the *r*-th component, satisfying ***S***_*r*_ = ***P***_*r*_***C***_0_. The expression of the Hill tensor ***P***_*r*_ of sphere inclusions can be found in the previous result^4^.

These isotropic stiffness parameters of LF and DF can be predicted by the MT method, which are demonstrated in Table 1. Overall, it can be expressed that the bulk modulus of LF is close to that of DF, while the shear modulus of LF is significantly higher than that of DF. It should be emphasized that the moduli in Table 1 are the dynamic elastic moduli^13^. The static moduli related to static mechanical properties of rock are obtained by the conversion of the dynamic elastic moduli, which are expressed as

8 $$ C_{{{\text{st}}}} = \alpha C_{{{\text{dyn}}}} , $$

where *C*_st_ represents the static elastic constant, *C*_dyn_ represents the dynamic elastic constant, and *α* is the coefficient. The quantity *α* of the Young’s modulus is taken as 0.39 in this study. Correspondingly, the Young’s moduli of LF and DF are 33.62 GPa and 26.64 GPa.

The compressive stiffness of LF and DF was determined by the uniaxial compressive strength of sandstone and soft rock obtained by He et al.^26^. The sandstone in this test contains 54% quartz, 25% clay, 13% dolomite, 2% albite, and 6% siderite. And the soft rock contains 36% quartz, 38% clay, 25% dolomite, and 1% siderite. The uniaxial compressive strength for the sandstone and the soft rock is 84.791 MPa or 16.766 MPa. Compared with the composition of these two kinds of rocks, the uniaxial compressive strength ratio between LF and DF can be set to approximately 4:1.

### Microscopic parameter calibration of each single phase

For the basic consensus on macroscopic and microscopic parameters of rock^9,40^, one has: (1) the effective modulus $\overline{E}^{*}$ of the parallel-bond element controls the macroscopic Young’s modulus under tension; (2) the effective modulus E^*^ of the linear contact element and $\overline{E}^{*}$ control the macroscopic Young’s modulus under compression; (3) the quantities κ^*^ and $\overline{\kappa }^{*}$ control the Poisson’s ratio; (4) the quantities $\overline{\sigma }_{{\text{c}}}$ and $\overline{\tau }_{{\text{c}}}$ control the macroscopic compressive strength (*σ*_c_); (5) the ratio of $\overline{\sigma }_{{\text{c}}}$ and $\overline{\tau }_{{\text{c}}}$ controls the failure mode of the sample. Thus, a series of homogeneous numerical samples (25 mm × 50 mm) containing 27,222 disc particles with the porosity being 0.1 were generated to explore the relationship between macroscopic and microscopic parameters.

The microscopic parameters of the rock example in help files of PFC^2D^ are used as the reference parameters in this calibration process. Afterwards, four groups of numerical tests with one uniaxial tensile test and three uniaxial compressive tests were conducted in sequence for the calibration of $\overline{E}^{*}$, E^*^, к^*^ and $\overline{\tau }_{{\text{c}}}$. Each previous calibration result serves as the input parameter of next calibration test. In order to simplify the influence of various microscopic parameters, the two parameter assumptions commonly using in DEM are expressed as

9 $$ \left\{ \begin{array}{*{20}l} \overline{\kappa }^{*} { = }\kappa \hfill \\ \overline{\sigma }_{{\text{c}}} = \overline{\tau }_{{\text{c}}} = \overline{c} \hfill \\ \end{array} \right., $$

which means $$\overline{\phi }{ = }0^\circ$$.

**Figure 7:**
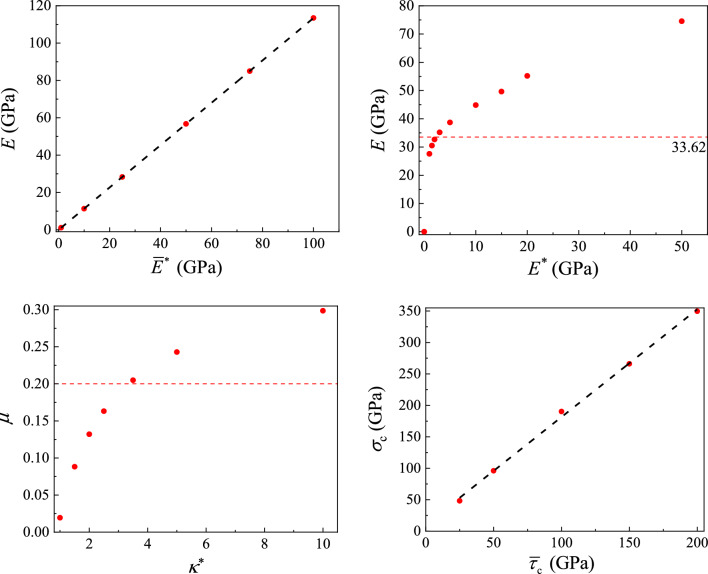
Macroscopic parameters calibration of LF.

The relationships between macroscopic and microscopic parameters by fitting of LF as an example are shown in Fig. 7, and the calibration results of LF and DF are summarized in Table 2. The macroscopic and microscopic parameters take a linear correlation trend as a whole. However, it is difficult to obtain satisfactory simulation results directly using the calibration results for subsequent simulations, probably due to the following reasons^34,42^. The first one is the lack of the tensile Young’s modulus test data, and E^*^ may be overestimated when the tensile Young’s modulus is considered equal to the compressive Young’s modulus. The other one is that the uniaxial compressive strength of LF and DF still differ from that of the sandstone and soft rock, respectively, because of different content and particle size of minerals. Therefore, on the basis of calibration parameters, the revised microscopic parameters can be obtained through repeated ‘trial and error’ and comparison with experimental results, which are listed in Table 2. In addition, the contact properties between LF and DF are set to be the same as those of DF.

**Table 2:** Microscopic parameters of calibration and revision.

Micro-parameters	LF (cal)	DF (cal)	LF (rev)	DF (rev)
E*/GPa	29.6	24.7	16.1	4.0
$\overline{E}^{*}$/GPa	2.7	2.0	16.1	4.0
$\overline{\kappa }^{*} { = }\kappa$	3.4	5.8	2.5	3.5
$\overline{\sigma }_{{\text{c}}}$/MPa	43.5	10.3	102	45
$\overline{c}$/MPa	43.5	10.3	102	45
$\overline{\phi }$/°	0	0	0	0

### Comparison between experimental and numerical results

**Figure 8:**
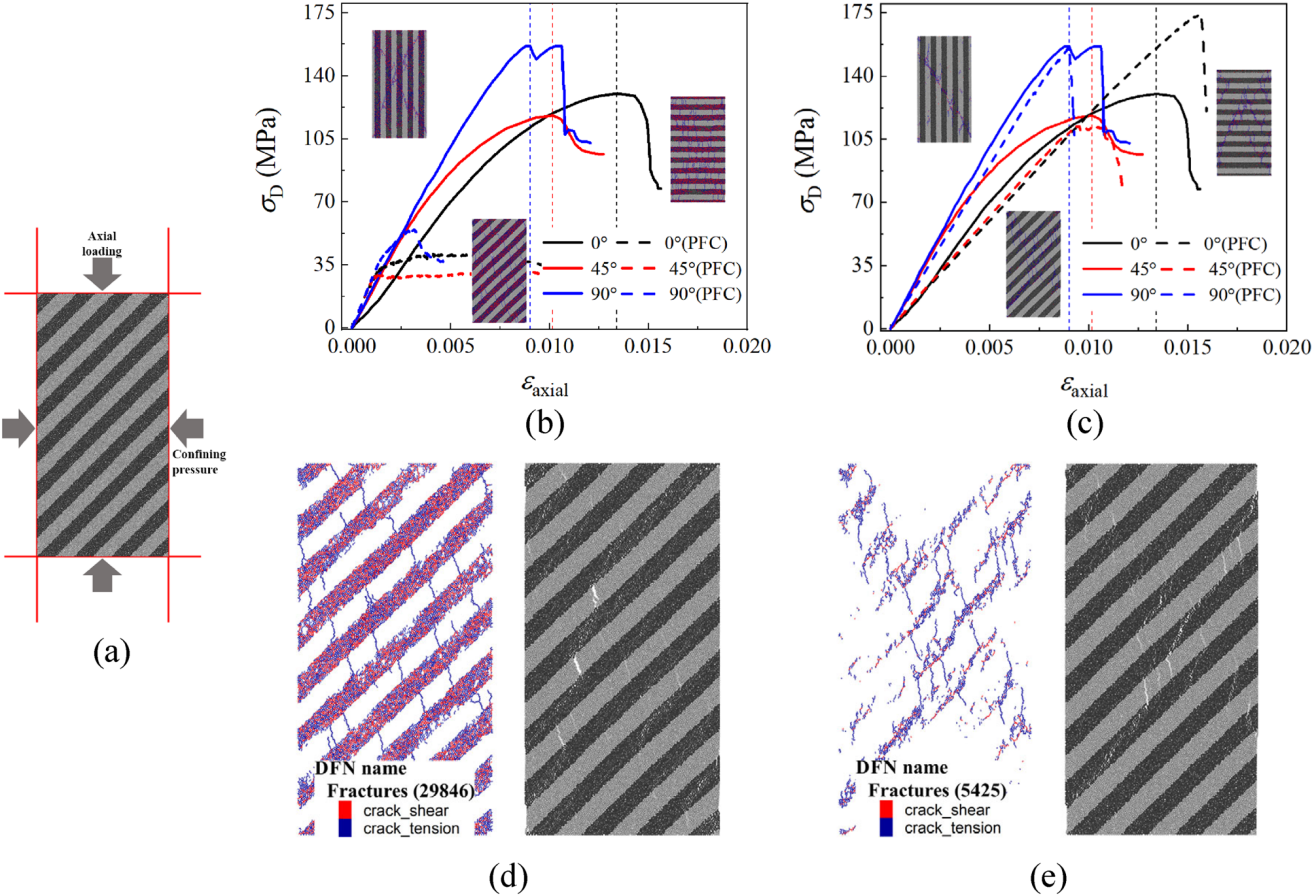
Comparison between calibration and revision microscopic parameters, where (**a**) is the loading mode of the numerical sample, (**b,c**) are the stress–strain curves with calibration and revision microscopic parameters, respectively, (**d,e**) are the number and distribution of cracks in 45° samples with calibration and revision microscopic parameters, respectively.

For this triaxial compression experiment, the final axial loading was carried out after various confining pressure loading paths^6^. Therefore, the confining pressures of 5 MPa is taken for simulation to comply with the impact of confining pressure on these experimental results. Figure 8 shows the simulation results with microscopic parameters of calibration and revision of the deviatoric stress–strain curve between experimental and numerical results. Herein, *σ*_D_ is the deviatoric stress, and *ε*_axial_ is the axial strain.

The method for simulation of axial loading and confining pressure is by applying stress and displacement loads to the corresponding walls in PFC, which is shown in Fig. 8a. And the axial loading in simulation will stops until the current stress is below 0.7 times the peak stress. It is clearly seen that simulated stress–strain relationship in use of the revised microscopic parameters were more consistent with the experimental results than that directly using calibrated microscopic parameters, which are shown in Fig. 8b,c, respectively. Figure 8d,e shows the micro-crack distribution of revision and calibration for the numerical 45° samples. As mentioned in “Microscopic parameter calibration of each single phase” section, directly using calibrated microscopic parameters may not predict the experimental results, and there may even be significant errors. Calibrated microscopic strength parameters are too low to increase the impact of confining pressure. The simulation results of 45° and 90° samples in Fig. 8c has significant plastic deformation and a large number of cracks are generated within the DF which are almost completely discrete.

Compared with the results of 45° and 90° samples in Fig. 8b, there is a significant strength error between the numerical and experimental results in the 0° sample. Through the past experience of layered materials^24,26,39^, it can be concluded that the peak stress of 0° sample in the laminated rock with complete structures is usually not much lower than that of the 90° sample. It is clearly observed in Fig. 3 that there are initial cracks in the 0° sample, while there are no obvious initial cracks in the 45° and 90° samples. Meanwhile, it can be analyzed from Fig. 8d that tensile cracks can be clearly identified in the sample after testing, which should be used to compare with the generated cracks in the CT slices. And the differences of crack characteristics between the 0° sample and the other two samples in Fig. 2 may be caused by the initial defects in the 0° sample.

**Figure 9:**
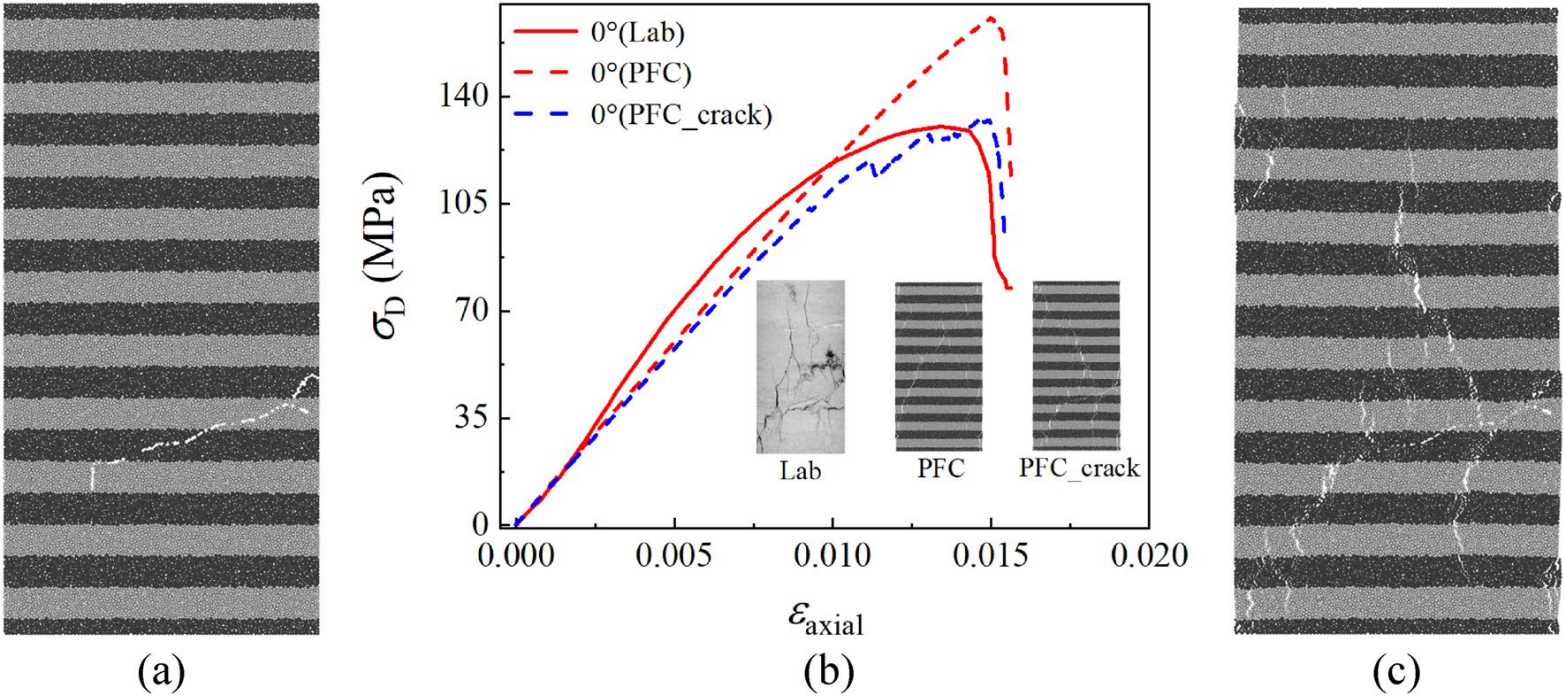
Simulation results of the 0° sample with initial main cracks, where (**a**) is the 0° numerical sample with initial cracks, (**b**) is the comparison among the experimental results, the ideal model and the crack containing model, and (**c**) is the failure mode of the 0° sample with initial cracks.

The initial main crack in the 0° sample is extracted by using the similar method in “Image processing of the shale sample slices” section, and the corresponding particles and contacts are removed within the main crack range. Figure 9 shows the 0° numerical sample with main cracks before compression, and the simulation results are compared with the experiments of the 0° sample in Fig. 8b. The initial crack significantly reduces the peak stress of the numerical sample, and the stress–strain curve is closer to the experimental results. In addition, the initial crack also changes the crack’s evolution form in numerical results, and this fact can explain the generation of the secondary cracks in the 0° sample CT slices. If there are no obvious initial defects in the 0° sample, the cracking mode of the 0° sample should be close to oblique cracking along the main crack similar to the 45° and 90° samples.

Futhermore, macroscopic mechanical parameters of samples obtained by the experimental and numerical results are compiled and listed in Table 3. All macroscopic parameters of both from the experiment and simulation demonstrate the same variation trend with the layered anlge, which means the revised microscopic parameters in Table 2 can be applied to analyze the mechanical behaviors of the laminated shale sample.

**Table 3:** Comparison of macroscopic parameters between experiment and simulation.

Angle	0°		45°		90°	
Method	Lab	PFC	Lab	PFC	Lab	PFC
*E*/GPa	12.79	11.02	17.55	12.43	20.02	18.12
*σ*_c_/MPa	130.13	132.74	117.97	111.80	156.47	154.91
*ε*_c_/10^–2^	1.34	1.47	1.02	0.94	0.90	0.90
Cracks						

## Numerical simulation on mechanical behaviors of the laminated shale

### Sensitivity analysis of microscopic mechanical parameters

**Figure 10:**
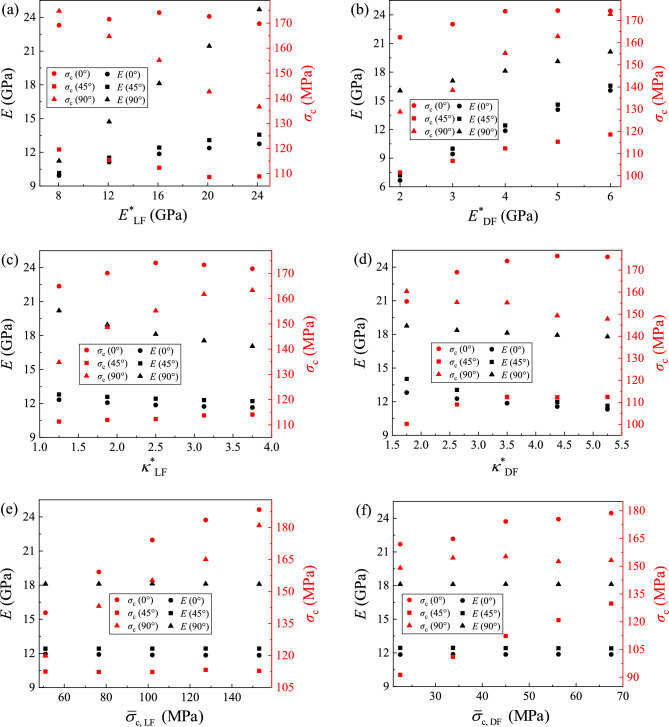

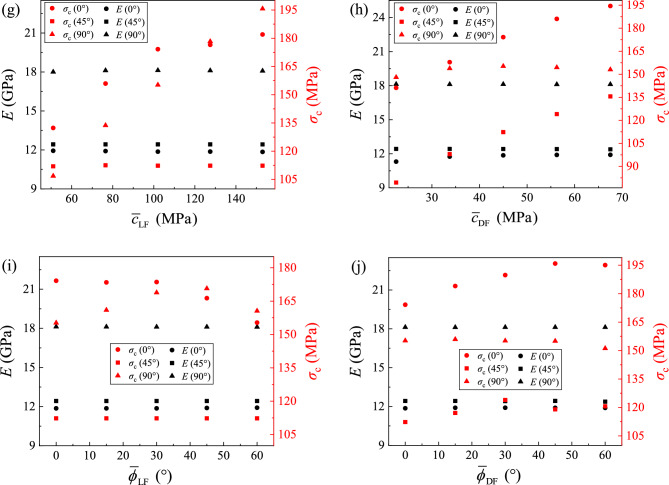
Relationship between macroscopic and microscopic mechanical parameters.

When the discrete element model contains two different mineral combinations (LF and DF), more microscopic parameters in the laminated model are required to be analyzed than that in the homogenous model. The superposition of microscopic parameters in this dual-model increases the complexity of the relationship between macroscopic and microscopic parameters. Therefore, the five key microscopic parameters containing $\overline{E}^{*} = E^{*}$, $\overline{\kappa }^{*} = \kappa^{*}$, $\overline{\sigma }_{{\text{c}}}$, $\overline{c}$ and $\overline{\phi }$ in DF and LF are set to 0.5, 0.75, 1, 1.25 and 1.5 times benchmark values of microscopic parameters in Table 2 (rev) for sensitivity analysis, where E^*^ and к^*^ are the microscopic stiffness parameters and $\overline{\sigma }_{{\text{c}}}$, $\overline{c}$ and $\overline{\phi }$ are the microscopic parameters related to strength. After analyzing the stress–strain curves of a total of 150 models in 10 groups, the effects of each microscopic parameters on the macroscopic parameters (*E* and *σ*_c_) of 0°, 45° and 90° sample are organized and expressed in Fig. 10. With the increasing of the microscopic parameters, the values of macroscopic *E* and *σ*_c_ exhibit different responsive rule.

It can be found from Fig. 10a–d that microscopic stiffness parameters not only affect the macroscopic stiffness parameters *E*, but also have an impact on the strength parameters *σ*_c_. With the increasing E^*^ in LF (E^*^_LF_) shown in Fig. 10a, E of samples decreases and *σ*_c_ of samples increases. Correspondingly, *E* and *σ*_c_ of samples increase with the increase of E^*^ in DF (E^*^_DF_) in Fig. 10b. By comparing Fig. 10a,b, it can be observed that E^*^_LF_ particularly affects *E* of the 90° sample whereas that E^*^_DF_ affects *E* of the 0° and 45° samples more significantly. In terms of strength, it is interesting that E^*^ significantly affects *σ*_c_ of the 90° sample, while *σ*_c_ of the 0° and 45° samples is also slightly affected by E^*^. Figure 10c,d shows the influence by the change of κ^*^ in LF and DF, respectively. Although κ^*^ usually affects the macroscopic Poisson’s ratio of the sample, it can be observed that it also affects *E* and *σ*_c_ of the samples. With the increase of κ^*^_LF_ in Fig. 10c, E of the 90° sample decreases, *σ*_c_ of the 90° sample increases, and macroscopic parameters of the other two samples have no significant change.

The effect of $\overline{\sigma }_{{\text{c}}}$, $\overline{c}$ and $\overline{\phi }$ in LF and DF on macroscopic parameters is shown in Fig. 10e–j. From these data, it can be seen that these microscopic strength parameters have no significant impact on the macroscopic stiffness (*E*) of samples. In terms of strength, $\overline{\sigma }_{{\text{c, LF}}}$ affects *σ*_c_ of the 0° and 90° samples (Fig. 10e) and $\overline{\sigma }_{{\text{c, DF}}}$ affected the *σ*_c_ of 0° and 45° samples (Fig. 10f). The value of $\overline{c}_{{{\text{LF}}}}$ affects *σ*_c_ of the 0° and 90° samples and $\overline{c}_{{{\text{DF}}}}$ affects *σ*_c_ of the 0° and 45° samples, and the laws are similar, but the degree of impact is not the same. Especially, *σ*_c_ of the 90° sample significantly increases with the increase of $\overline{c}$. For the influence of $\overline{\phi }$ in Fig. 10i,j, $\overline{\phi }$ has an irregular effect on *σ*_c_. Although $\overline{\phi }$ can increase the microscopic shear strength $\overline{\tau }_{{\text{c}}}$, $\overline{\phi }$ does not have a significant impact on *σ*_c_ of sample as $\overline{\sigma }_{{\text{c}}}$ and $\overline{c}$.

### Effect of the component distribution

We then discuss the influence of microstructures of laminated shale on the simulation. The CT images obtained in “Establishment of the numerical sample” section are used to establish numerical models that are closer to the actual situation than the ideal model. The microscopic parameters used for simulation are taken from Table 2.

**Figure 11:**
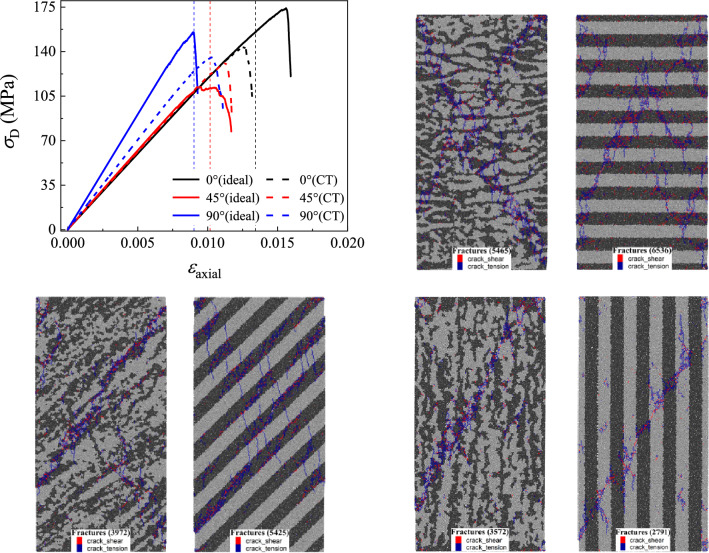
Comparison between the ideal and the CT models.

The comparison of the CT model with the ideal model is shown in Fig. 11, and it can be seen that there is a significant difference in the stress–strain curves between the two models. Although the microscopic parameters are the same, it is surprising that the difference in microstructures has a significant impact on the numerical specimens. The anisotropic patterns of the CT model is consistent with that of the ideal model. That is, the value of *σ*_c_ for the 0° sample is the biggest, and that of the 45° is the smallest. The value of *E* for the 90° sample is bigger than those of the other two samples, as they also have the same value. However, the anisotropic degree of the CT model is lower than that of the ideal model. For microstructures, the ideal model is the most anisotropic model, and the component arrangement in the CT model is not strictly an ideal layered structure. For ideal models of 0° and 90° samples, the through cracks during failure need to penetrate the LF components, so the strength of the ideal model is relatively high. However, the ideal model of the 45° sample is prone to generating oblique main cracks inside the DF, resulting in a lower value of *σ*_c_. For the CT models, cracks tend to evolve more along irregular paths in the DF, resulting in the lower *σ*_c_ of the 0° and 90° samples compared to the ideal model, while *σ*_c_ of the 45° sample is higher than that of the ideal model.

**Figure 12:**
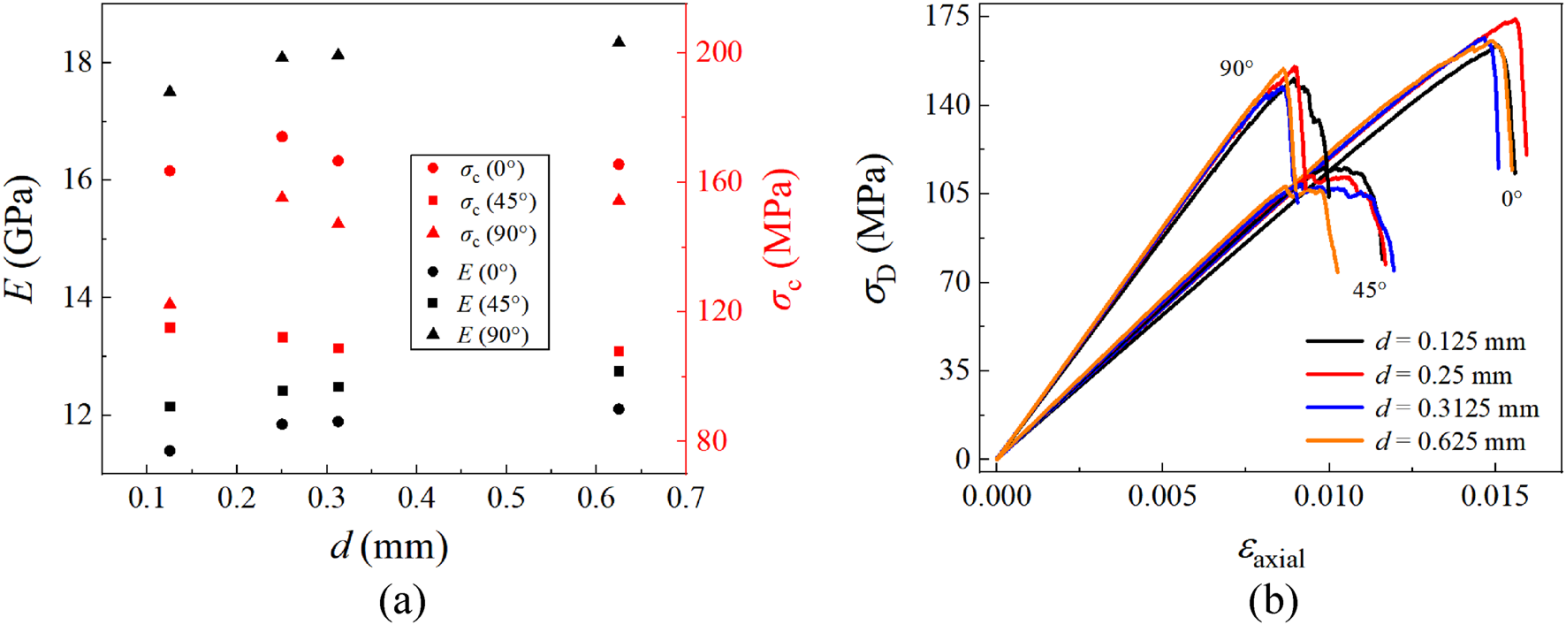
The influence of layered thickness on ideal models.

In addition to the irregular distribution of components, the layer thickness (*d*) is also an important parameter for component distribution. Four sets of ideal models with different thicknesses are used to analyze the effect of *d* on the mechanical properties of specimens, with the ratio between LF and DF still being 1:1. It can be seen from Fig. 12a that *E* increases with the increase of *d*. The value of *σ*_c_ of the 45° sample slightly decreases with the increase of *d*. The values of *σ*_c_ of the 0° and 90° samples manifest an irregular effect on *d*, and the maximum reaches when *d* = 0.25 mm. By comparing Figs. 11 and 12, it can be found that the influence of layer thickness on macroscopic mechanical properties is not as significant as that of the irregular distribution of components (CT), which means that the impact of the irregular distribution of components requires further optimization.

### Effect of different confining pressures

**Figure 13:**
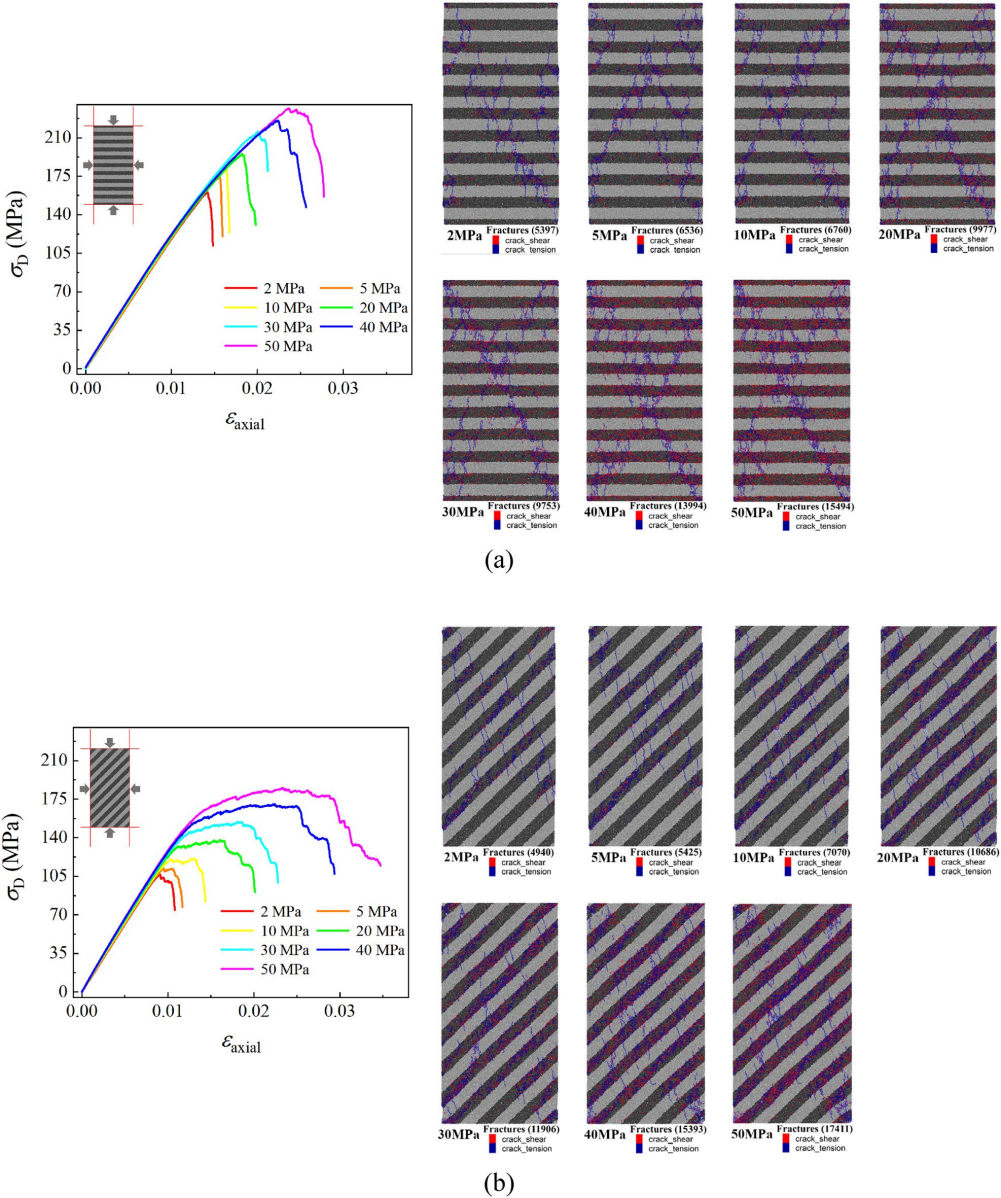

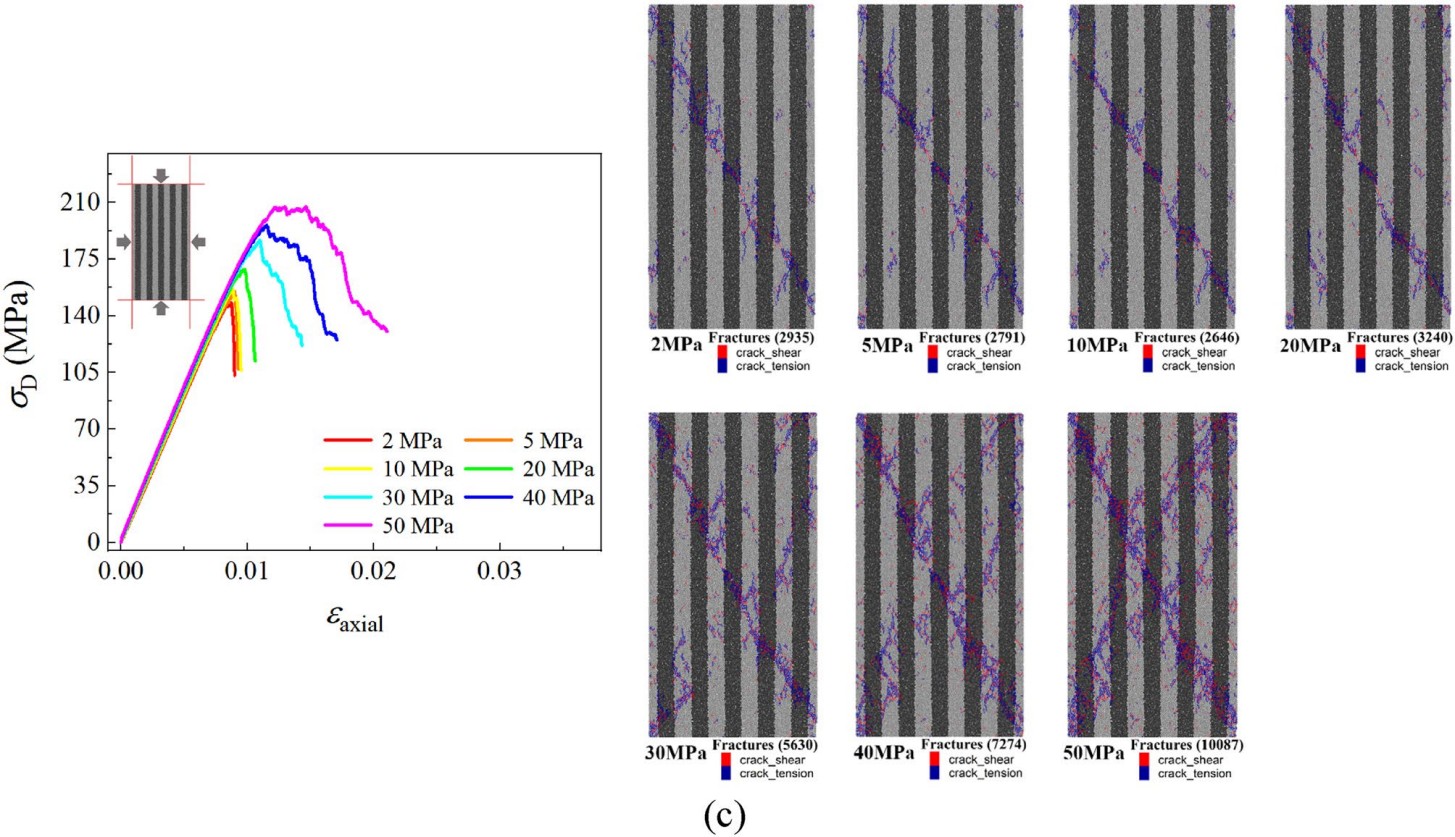
The stress–strain curve and crack number distribution of the 0°, 45° and 90° samples under different confining pressure values.

In the experiment, the confining pressure is an important factor that affects the mechanical behaviors of shale especially in deep and ultra-deep reservoirs. As a result, the stress–strain curves of three kinds of layered angle samples under different confining pressures are obtained by changing the confining pressure of the numerical sample under revised microscopic parameters in Table 2, which are shown in Fig. 13. From the stress–strain curve of the three kinds of samples, it can be seen that the confining pressure significant increases the yielding and peak stress of the samples, but has no significant effect on the stiffness of the three samples. Figure 13 also shows the number and distribution of cracks in three sets of samples after failure of samples. It can be seen that the number of cracks in the failure sample increases with the increase of the confining pressure, and the main crack of samples changes from the oblique main crack to the ‘X-shaped’ main crack with the increase of the confining pressure.

In addition, there are some different patterns in the 0°, 45°, and 90° samples with the increasing confining pressure, and some unique properties only occur in certain samples. In the elastic stage of the stress–strain curve, the tangential modulus of the 0° sample exhibits a slight decrease during the loading process, which becomes apparent as the confining pressure increases. Then, after the elastic stage, the failure mode of the sample gradually changes from brittleness to ductility with the increase of the confining pressure, which is particularly evident in the 45° sample, and there are even plastic deformation features which appear in the 45° samples. Meanwhile, this plastic property is not evident in the 0° and 90° samples. After the sample reaches the peak stress and then it is destructed, the sample exhibits a certain residual strength that increases with the confining pressure.

## Conclusions

In conclusion, a discrete element numerical sample is established to match the laminated shale, and the microstructures and mineral compositions of the laminated shale based on CT images provided by digital cores are fully considered in the modeling process. The mechanical behaviors of the shale sample are simulated via the PFC^2D^, which are in accordance with the experimental results. The results show that, the laminated shale can be simplified as a laminated composite material containing both light facies (LF) and dark facies (DF) by observing CT images. The discrete element model with ideal layered structures can still simulate the macroscopic anisotropic mechanical behaviors of the laminated shale when the microscopic parameters of the LF and DF are fully considered. Through the analysis of the CT images and comparison of the experimental and simulated results, it was found that the initial cracks are the key to the difference between the experimental and simulated results. The Initial cracks lead to decrease the compressive strength of the 0° sample, and they can change the failure mode of the 0° sample. For the two-phase composite material when analyzing the laminated shale, the microscopic strength parameters of the parallel bonding model mainly affect these macroscopic strength parameters, while the microscopic stiffness parameters have a significant impact on both the macroscopic stiffness and strength parameters. The degree of these impacts is related to the layering angle and the face type. The confining pressure significantly affects the strength of the laminated shale, and the 45° sample exhibits obvious plastic properties as the confining pressure increases. In addition, the microstructure difference between the ideal and the CT model considerably influence the macroscopic mechanical behaviors of the laminated samples, and the microstructures of the ideal model have a stronger anisotropy than that of the CT model.

The above analyses help to improve understanding of the anisotropic mechanical properties of the laminated shale, and they can provide the guidance and inspirations for corresponding simulation techniques. The sensitivity analysis shows that variations in microscopic parameters can lead to substantial changes in stiffness and strength, which are crucial for designing and optimizing hydraulic fracturing processes. Understanding the impact of confining pressure on mechanical properties helps in predicting the behavior of shale reservoirs under different stress conditions, enhancing the efficiency and safety of shale gas extraction operations.

### Informed consent

Written informed consent for publication of this paper was obtained from the China University of Petroleum and all authors.

## Data Availability

The supporting information involved in this study is available within this article.
